# Brain infarctions after glioma surgery: prevalence, radiological characteristics and risk factors

**DOI:** 10.1007/s00701-021-04914-z

**Published:** 2021-09-01

**Authors:** Per S. Strand, Erik M. Berntsen, Even H. Fyllingen, Lisa M. Sagberg, Ingerid Reinertsen, Sasha Gulati, David Bouget, Ole Solheim

**Affiliations:** 1 Department of Neuromedicine and Movement Science, Norwegian University of Science and Technology, Trondheim, Norway; 2 Department of Neurosurgery, St. Olavs Hospital, Trondheim University Hospital, Trondheim, Norway; 3 Department of Radiology and Nuclear Medicine, St. Olavs Hospital, Trondheim University Hospital, Trondheim, Norway; 4 Department of Circulation and Medical Imaging, Faculty of Medicine and Health Sciences, Norwegian University of Science and Technology, Trondheim, Norway; 5 Department of Public Health and Nursing, Faculty of Medicine and Health Sciences, Norwegian University of Science and Technology, Trondheim, Norway; 6 Department of Health Research, SINTEF Digital, Trondheim, Norway

**Keywords:** Glioma, Surgery, Infarction, Diffusion-weighted imaging

## Abstract

**Background:**

Prevalence, radiological characteristics, and risk factors for peritumoral infarctions after glioma surgery are not much studied. In this study, we assessed shape, volume, and prevalence of peritumoral infarctions and investigated possible associated factors.

**Methods:**

In a prospective single-center cohort study, we included all adult patients operated for diffuse gliomas from January 2007 to December 2018. Postoperative infarctions were segmented using early postoperative MRI images, and volume, shape, and location of postoperative infarctions were assessed. Heatmaps of the distribution of tumors and infarctions were created.

**Results:**

MRIs from 238 (44%) of 539 operations showed restricted diffusion in relation to the operation cavity, interpreted as postoperative infarctions. Of these, 86 (36%) were rim-shaped, 103 (43%) were sector-shaped, 40 (17%) were a combination of rim- and sector-shaped, and six (3%) were remote infarctions. Median infarction volume was 1.7 cm^3^ (IQR 0.7–4.3, range 0.1–67.1). Infarctions were more common if the tumor was in the temporal lobe, and the map shows more infarctions in the periventricular watershed areas. Sector-shaped infarctions were more often seen in patients with known cerebrovascular disease (47.6% vs. 25.5%, p = 0.024). There was a positive correlation between infarction volume and tumor volume (r = 0.267, p < 0.001) and infarction volume and perioperative bleeding (r = 0.176, p = 0.014). Moreover, there was a significant positive association between age and larger infarction volumes (r = 0.193, p = 0.003). Infarction rates and infarction volumes varied across individual surgeons, p = 0.037 (range 32–72%) and p = 0.026.

**Conclusions:**

In the present study, peritumoral infarctions occurred in 44% after diffuse glioma operations. Infarctions were more common in patients operated for tumors in the temporal lobe but were not more common following recurrent surgeries. Sector-shaped infarctions were more common in patients with known cerebrovascular disease. Increasing age, larger tumors, and more perioperative bleeding were factors associated with infarction volumes. The risk of infarctions and infarction volumes may also be surgeon-dependent.

## Introduction

The prognosis of diffuse glioma improves with extent of surgical resection [19, 16, 20], but glioma surgery is a balance between extensive tumor resections and avoiding damage to adjacent functional brain tissue. Based on early postoperative magnetic resonance imaging (MRI) with diffusion-weighted imaging (DWI), it has been reported that perioperative and mostly peritumoral infarctions occur in 19–80% of patients undergoing tumor surgery. These infarctions have been associated with postoperative neurological deficits and impaired function [8, 9, 17, 21, 15].

However, characteristics and risk factors for peritumoral infarctions are still not much explored, although some but not all studies report higher risks of infarctions following reoperations [8, 5].

The location of a cerebral infarction is critical for the neurological outcome [13]. In a study of 177 diffuse glioma procedures, it was reported that new postoperative DWI lesions occurred more often in the insula, the operculum, and the temporal lobe [5]. Furthermore, a small study including eleven patients with opercular tumors found that at least nine patients had infarctions in relation to the resection cavity [10]. However, a larger study including 109 patients found no correlation between tumor location and incidence of acquired ischemic lesions, where only a close relation to central arteries was found to be a significant risk factor [8]. Thus, it is still unclear if tumor location in certain brain areas is associated with risk of postoperative ischemic lesions.

In the present study, we sought to assess the shape, volume, and prevalence of peritumoral infarctions, and investigate possible patient- or tumor-related risk factors, including tumor location based on a population-based patient selection and manual volumetric segmentations of postoperative DWI changes.

## Methods

### Patients and clinical data

We screened all adult patients (≥ 18 years) operated for newly diagnosed or recurrent diffuse gliomas WHO grades 2–4 at the Department of Neurosurgery at St. Olavs Hospital, Trondheim University Hospital, from January 2007 through December 2018, with available postoperative MRI including DWI performed within 72 h after surgery. This department exclusively serves approximately 750, 000 inhabitants in a defined geographical catchment region.

Patients operated before the second half of 2016 were classified by a neuropathologist according to the 2007 WHO classification of central nervous system-tumors [11], whereas gliomas operated in the latter half of 2016 through 2018 were classified according to the 2016 WHO classification [12].

Clinical data were collected from electronic medical records in a local tumor registry. The patients’ physical status was assessed by an anesthesiologist prior to surgery, using the American Society of Anesthesiologists Classification (ASA). Karnofsky performance status (KPS) was rated by the operating surgeon just prior to surgery, using a questionnaire. Missing clinical data were retrospectively assessed and collected from electrical medical records from all seven hospitals in our catchment region.

### MRI scans and DWI analyses

The early postoperative MRI scans consisted of pre-contrast T1, T2, FLAIR, DWI sequences, and a post-contrast T1. DWI was performed according to clinical routine with a standard echo-planar imaging sequence (EPI), and apparent diffusion coefficient (ADC) maps were automatically calculated. Areas with high signals on B1000 images and corresponding low values on ADC maps were considered to have restricted diffusion representing cytotoxic edema following acute ischemia, as long as the anatomical images showed no other explanation, and the relative ADC value (rADC) was lower than 0.7 × 10^−3^ mm^2^/s compared to the same area in the contralateral hemisphere. This cutoff value was based on previous studies exploring the time course of ADC map changes in brain ischemia [6]. To exclude diffusion abnormalities related to blood products, areas with high signal on B1000 images that could not clearly be distinguished from areas with high signal on pre-contrast T1 were classified as non-ischemic. Furthermore, preoperative MRI images were also reviewed to exclude other possible sources for the diffusion changes, for instance, likely residual tumor, artifacts, or abscesses.

A medical student trained by an experienced neuroradiologist manually segmented areas with postoperative ischemia using 3D-Slicer version 4.9.0 (3D_Slicer, Boston Massachusetts) based on the areas with high signal on the B1000 changes. In cases of doubt, an experienced neuroradiologist was consulted. Tumors were either semi-automatically or manually segmented from preoperative MRI scans. Non-enhancing or partially enhancing tumors were segmented in 2D or 3D FLAIR volumes, whereas contrast-enhancing tumors were segmented in contrast-enhanced 3D T1 images. Tumor volume segmentations were validated by an experienced neurosurgeon or experienced neuroradiologist.

**Fig. 1:**
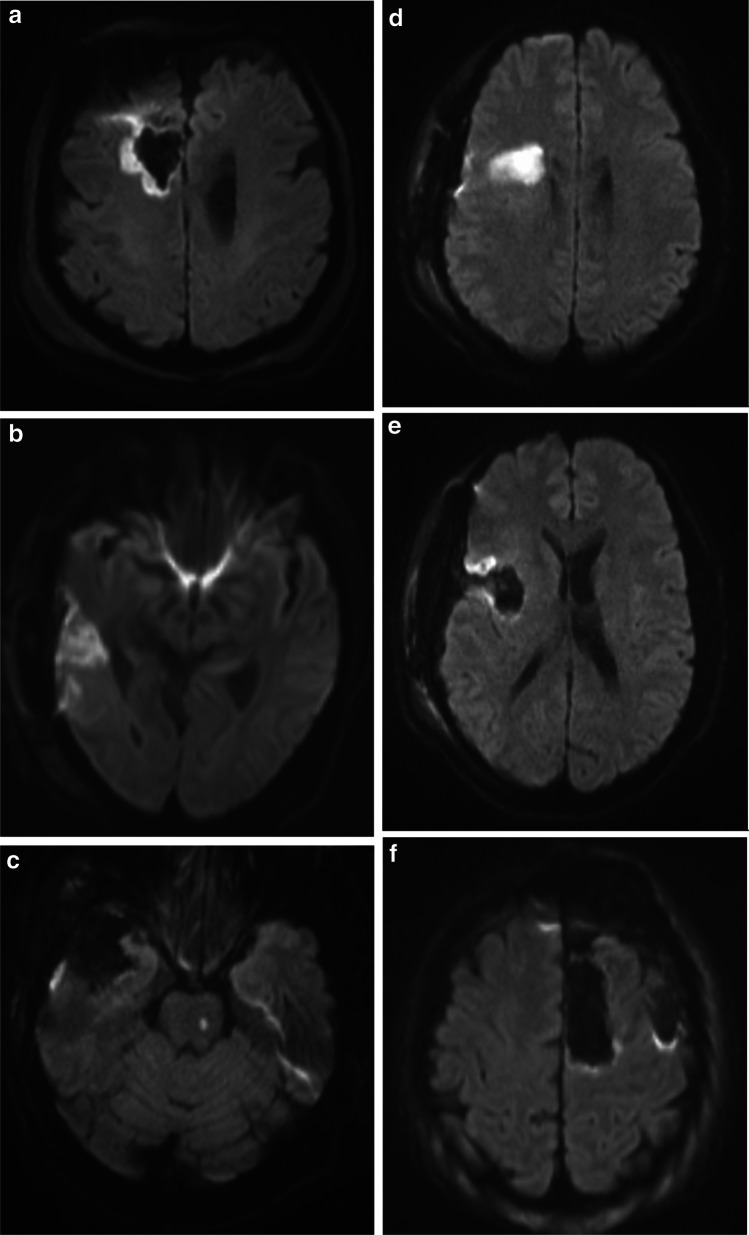
**A** Rim-shaped DWI change (volume = 2.7 cm^3^). **B** Sector-shaped DWI change (volume = 2.5 cm^3^). **C** A remote pontine DWI-change (volume = 0.2 cm^3^). **D** and **E** A sector-shaped and rim-shaped DWI change in the same brain (volume = 5.4 cm^3^). **F** A cavity without any significant DWI abnormality

As described in a previous publication from our group [9], DWI abnormalities were classified as either rim (lesions surrounding the cavity), sector, combined (combination of rim and sector), or remote infarctions (not abutting the resection cavity). Minimal areas of increased signal on DWI images in relation to the operation cavity are commonly seen [15, 22]. We therefore used a 3-mm radial diameter cutoff to separate these unspecific postoperative signal changes from radiological significant rim-shaped infarctions (Fig. 1).

Maps of the tumor distribution and infarctions were created based on preoperative tumor segmentations and postoperative infarction segmentations, respectively. All the MR images and corresponding segmentations were spatially aligned with a pre-defined brain template from the Montreal Neurological Institute (MNI—ICBM-152 average brain) [7]. To perform this alignment, the advanced normalization tools framework (ANTs) [1], and more specifically the symmetric diffeomorphic method (SyN), was used. Either the T1 or FLAIR MNI atlas was used to register the preoperative MRI volumes, and the T2 atlas was selected for registering the DWI volumes. A custom and deep learning-based skull-stripping approach has been favored over the built-in approach from the framework. The architecture used is a regular 3D U-Net [23], trained over 300 samples of mixed T1 and FLAIR MRI volumes, and the implementation was done in Python using Keras and Tensorflow. The resulting registration transformations were applied to the individual segmentations to merge all the tumors and infarctions into their common space, yielding the final maps.

### Statistical analyses

Statistical analyses were performed with IBS SPSS Statistics version 25.0 (IBM, Armonk, New York). Kolmogorov–Smirnov test and Q-Q-plots were used to determine normal distribution of data. Differences between groups were assessed using independent samples *t* tests and Pearson’s chi-square tests, for continuous and categorical variables, respectively. Mann–Whitney *U* and Kruskal–Wallis tests were used for non-parametric data. Spearman’s rank correlation test was used to assess correlation between two continuous variables. Statistical significance level was set to p ≤ 0.05.

### Ethics and approval

The study protocol was approved by the Regional Ethical Committee for Health Region Mid-Norway (REK), (REK reference 2018/1187). All patients provided written informed consent (REK reference 2011/974). The data collection was done according to the guidelines of the Helsinki Declaration.

## Results

**Fig. 2:**
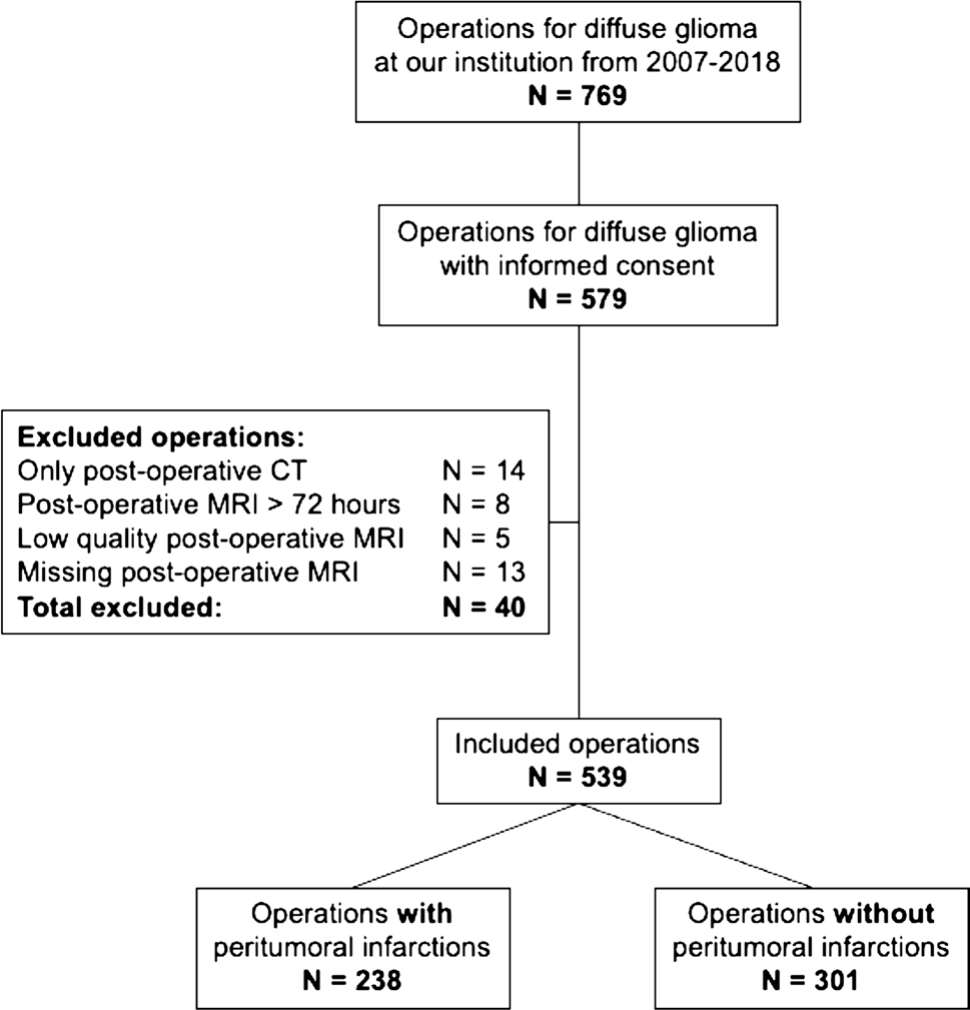
A flowchart of the inclusion process

A flowchart of the inclusion process is shown in Fig. 2. Out of 769 eligible operations, 579 gave informed consent to be included in research. Early postoperative MRIs were available following 539 procedures, where 320 were primary operations and 219 were reoperations.

Restricted diffusion in postoperative MRI DWI scans interpreted as infarctions were found in 238 (44%) of the 539 operations, while 301 of 539 (56%) of the postoperative MRI scans had no significant DWI signal changes. Of the 238 infarctions, 86 (36%) were rim-shaped, 103 (43%) were sector-shaped, 40 (17%) were a combination of rim- and sector-shaped, and six (3%) were remote infarctions. In patients with infarctions, the median infarction volume was 1.7 cm^3^ (inter-quartile range [IQR] 0.7–4.3, range 0.1–67.1). The median infarction volume for sector-shaped or combined rim- and sector-shaped infarctions was significantly larger than operations with rim infarctions alone, 2.4 cm^3^ vs 1.1 cm^3^ (p < 0.001). Four out of the six remote infarctions were in patients with glioblastomas, and two were in patients with diffuse low-grade gliomas. Median tumor volume in these six patients was 25.4 ml (IQR 5.6–34.4).

**Fig. 3:**
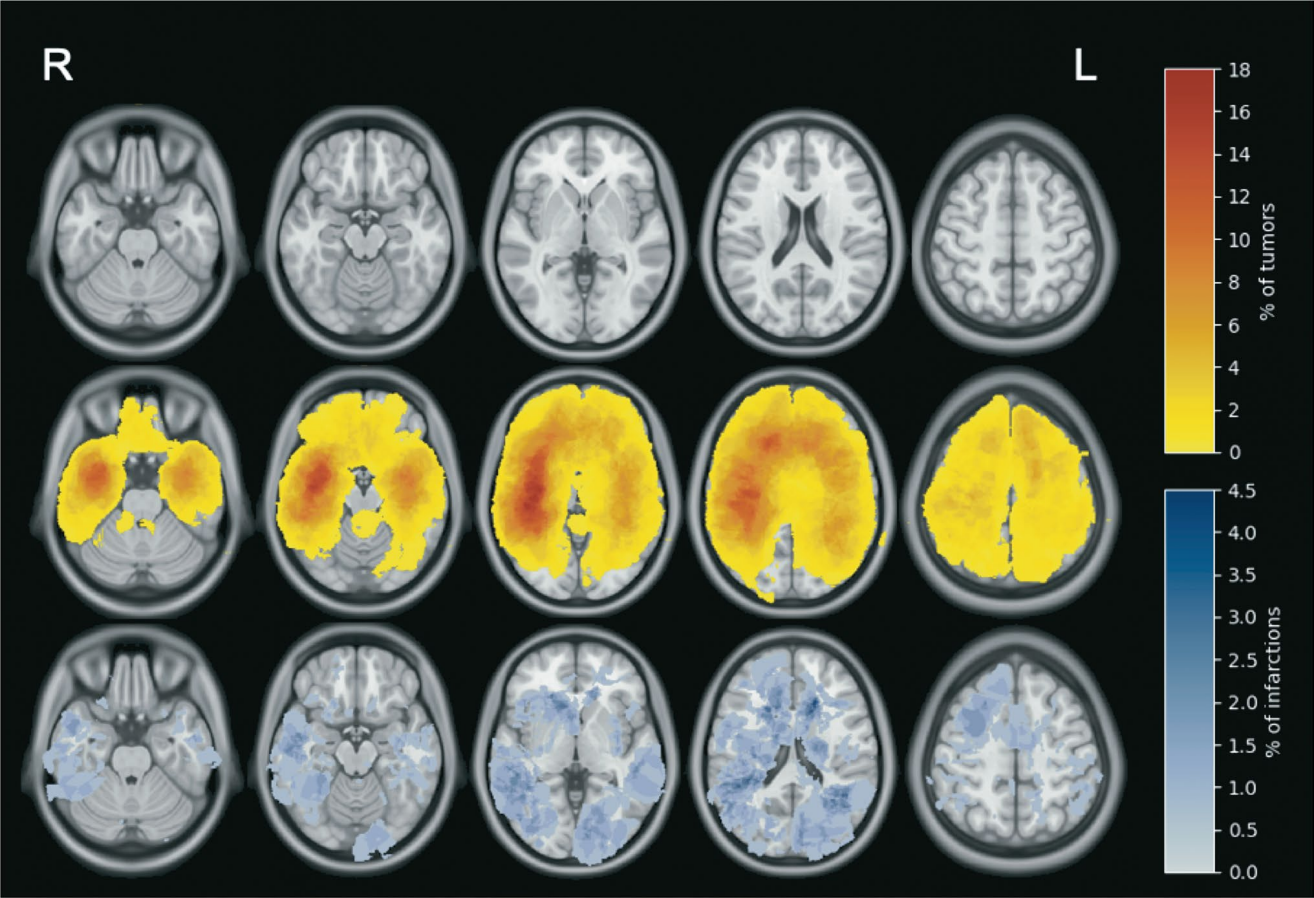
From top: a brain model for reference, a heatmap with the distribution of tumors, and a heatmap with the distribution of all infarctions. Areas of interest are presented in percentages

Characteristics of operations with and without postoperative significant DWI signal changes are presented in Table 1. As seen, peritumoral infarctions were more common in patients operated for tumors in the temporal lobe. We did not find any significant associations between postoperative infarctions and WHO grade, tumor lateralization, duration of surgery, ASA grade, preoperative KPS, previous radiotherapy or chemotherapy, or if the surgeon was a resident or consultant. The heatmap presented in Fig. 3 depicts the temporal predominance and shows a possible increased number of infarctions around the horns of the lateral ventricles.

**Table 1:** Characteristics of operations with vs. without peritumoral infarctions

Table 1	Any infarction	No infarction	p value
Number of operations	238 (44.2%)	301 (55.8%)	
Tumor volume in cm^3^ (IQR)	22.5 (8.5–49.8)	24.99 (9.4–48.3)	0.910
Age in quartiles			
1: range (18–44)	74 (51.4%)	70 (48.6%)	0.114
2: range (46–55)	57 (45.2%)	69 (54.8%)	
3: range (56–64)	60 (42.3%)	82 (57.7%)	
4: range (≥ 65)	47 (37.0%)	80 (63.0%)	
Sex			
Female	95 (44.4%)	119 (55.6%)	0.928
Male	143 (44.0%)	182 (56.0%)	
Tumor entity			
WHO grade 2	50 (48.1%)	54 (51.9%)	0.121
WHO grade 3	50 (48.5%)	53 (51.5%)	
WHO grade 4	132 (40.7%)	192 (59.3%)	
Unspecified LGG	3 (60.0%)	2 (40%)	
Unspecified HGG	3 (100%)	0 (0.0%)	
LGG (WHO grade 2)	53 (48.6%)	56 (51.4%)	0.293
HGG (WHO grades 3–4)	185 (43.0%)	245 (56.0%)	
Tumor lateralization			
Right	120 (47.6%)	132 (52.4%)	0.312
Left	104 (40.9%)	150 (59.1%)	
Bilateral/midline involvement	14 (42.4%)	19 (57.6%)	
Tumor distribution^*^			
Frontal	113 (40.5%)	166 (59.5%)	0.077
Temporal	102 (50.7%)	99 (49.3%)	**0.017**
Occipital	23 (44.2%)	29 (55.8%)	0.991
Parietal	50 (39.4%)	77 (60.6%)	0.214
Deep brain	27 (39.1%)	42 (60.9%)	0.368
Cerebellum	3 (50.0%)	3 (50%)	0.772
Brain stem	1 (25.0%)	3 (75.0%)	0.439
Intraventricular	0(0.0%)	1 (100%)	0.382
Operation			0.513
Primary surgery	145 (45.3%)	175 (54.7%)	
Reoperation	93 (42.5%)	126 (57.5%)	
Previous radiotherapy			0.316
Yes	67 (47.9%)	73 (52.1%)	
No	171 (30.1%)	398 (69.9%)	
Previous chemotherapy			0.566
Yes	46 (46.0%)	54 (54%)	
No	192 (43.7%)	247 (56.3%)	
Diabetes type 1 or 2			0.507
Yes	15 (50.0%)	15 (50.0%)	
No	223 (43.8%)	286 (56.2%)	
Cerebrovascular disease			0.222
Yes	12 (57.1%)	9 (42.9%)	
No	226 (43.6%)	292 (56.4%)	
ASA grade			0.542
1–2	174 (43.4%)	227 (56.6%)	
3–4	64 (46.4%)	74 (53.6%)	
Preoperative KPS			0.166
≥ 70	210 (45.3%)	253 (54.6%)	
< 70	28 (36.8%)	48 (63.2%)	
Surgeon			0.298
Resident	34 (39.1%)	53 (60.9%)	
Consultant	204 (45.1%)	248 (54.9%)	

^*^If the tumor was in multiple lobes, it is registered in all affected lobes

There was a positive correlation between infarction volume and tumor volume (r = 0.267, p < 0.001) and a positive association between infarction volume and intraoperative bleeding (r = 0.176, p = 0.014). Further, there was a significant positive association between age and infarction volumes (r = 0.193, p = 0.003) We found no correlation between infarction volume and the duration of the operations (r = 0.090. p = 0.170).

As a post-hoc analysis, we found a statistically significant difference in infarction rates and infarction volume across individual surgeons, p = 0.037 (range 32–72%) and p = 0.026 (range median 0.3–3.4 cm^3^), respectively. Subgroup analyses for rim-shaped and sector-shaped infarctions are presented in Tables 2 and 3, respectively. As seen, younger age was associated with more rim-shaped infarctions, while tumors in the temporal lobe and known cerebrovascular disease were associated with sector-shaped infarctions.

**Table 2:** Characteristics of operations with vs. without rim-shaped infarctions. Statistically significant values are highlighted

Table 2	Rim only	No rim infarction	p value
Number of operations	86 (16.0%)	453 (84.0%)	
Tumor volume in cm^3^ (IQR)	28.9 (9.3–49.3)	22.5 (8.9–48.7)	0.397
Age in quartiles			
1: range (18–44)	33 (22.9%)	111 (77.1%)	**0.018**
2: range (45–55)	22 (17.5%)	104 (82.5%)	
3: range (56–64)	19 (13.4%)	123 (86.6%)	
4: range (≥ 65)	12 (9.4%)	115 (90.6%)	
Sex			
Female	38 (17.8%)	176 (82.2%)	0.354
Male	48 (14.8%)	277 (85.2%)	
Tumor entity			
WHO grade 2	20 (19.2%)	84 (80.8%)	0.545
WHO grade 3	19 (18.4%)	84 (81.6%)	
WHO grade 4	42 (13.0%)	282 (87.0%)	
Unspecified LGG	1 (20.0%)	4 (80%)	
Unspecified HGG	1 (33.3%)	2 (67.7%)	
LGG (WHO grade 2)	21 (19.3%)	88 (80.7%)	0.210
HGG (WHO grades 3–4)	62 (14.4%)	368 (85.6%)	
Tumor lateralization			
Right	41 (16.3%)	211 (83.7%)	0.824
Left	41 (16.1%)	213 (83.9%)	
Bilateral/midline involvement	4 (12.1%)	29 (87.9%)	
Tumor distribution^*^			
Frontal	43 (15.4%)	236 (84.6%)	0.721
Temporal	31 (15.4%)	170 (84.6%)	0.795
Occipital	5 (9.6%)	47 (90.4%)	0.189
Parietal	22 (17.3%)	105 (82.7%)	0.630
Deep brain	8 (11.6%)	61 (88.4%)	0.289
Cerebellum	0 (0.0%)	6 (100%)	0.283
Brain stem	0 (0.0%)	4 (100%)	0.382
Intraventricular	0(0.0%)	1 (100%)	0.681
Operation			
Primary surgery	59 (18.4%)	261 (81.6%)	0.057
Reoperation	27 (12.3%)	192 87.7%)	
Previous radiotherapy			
Yes	21 (15.0%)	119 (85.0%)	0.712
No	65 (16.3%)	333 (83.7%)	
Previous chemotherapy			
Yes	12 (12.0%)	88 (88.0%)	0.666
No	74 (16.9%)	365 (83.1%)	
Diabetes type 1 or 2			0.099
Yes	8 (26.7%)	22 (73.3%)	
No	78 (15.3%)	431 (84.7%)	
Cerebrovascular disease			0.412
Yes	2 (9.5%)	19 (90.5%)	
No	84 (16.2%)	434 (83.8%)	
ASA grade			
1–2	66 (16.5%)	335 (83.5%)	0.586
3–4	20 (14.5%)	118 (85.5%)	
Preoperative KPS			
≥ 70	10 (13.2%)	66 (86.8%)	0.472
< 70	76 (16.4%)	387 (83.6%)	
Surgeon			
Resident	13 (14.9%)	74 (85.1%)	0.778
Consultant	73 (16.2%)	379 (83.8%)

**Table 3:** Characteristics of operations with vs. without sector infarctions

Table 3	Sector or rim + sector	No sector infarction	p value
Number of operations	142 (26.3%)	397 (73.7%)	
Tumor volume in cm^3^ (IQR)	20.4 (8.2–1.7)	25.4 (9.3–7.8)	0.463
Age in quartiles			
1: range (18–44)	37 (25.7%)	107 (74.3%)	0.994
2: range (45–55)	34 (27.0%)	92 (73.0%)	
3: range (56–64)	38 (26.8%)	104 (73.2%)	
4: range (≥ 65)	33 (26.0%)	94 (74.0%)	
Sex			
Female	53 (24.8%)	161 (75.2%)	0.500
Male	87 (26.8%)	238 (73.2%)	
Tumor entity			
WHO grade 2	29 (27.9%)	75 (72.1%)	0.388
WHO grade 3	31 (30.1%)	72 (69.9%)	
WHO grade 4	79 (24.4%)	245 (75.6%)	
Unspecified LGG	1 (20.0%)	4 (80%)	
Unspecified HGG	2 (66.7%)	1 (33.3%)	
LGG (WHO grade 2)	30 (27.5%)	79 (72.5%)	0.754
HGG (WHO grades 3–4)	112 (26.0%)	318 (74.0%)	
Tumor lateralization			
Right	74 (29.4%)	178 (70.6%)	0.328
Left	60 (23.6%)	194 (76.4%)	
Bilateral/midline-involvement	8 (24.2%)	25 (75.8%)	
Tumor distribution^*^			
Frontal	65 (23.3%)	214 (76.7%)	0.096
Temporal	67 (33.3%)	134 (66.7%)	**0.005**
Occipital	17 (32.7%)	35 (67.3%)	0.274
Parietal	27 (21.3%)	100 (78.7%)	0.137
Basal ganglia	17 (24.6%)	52 (75.4%)	0.730
Cerebellum	2 (33.3%)	5 (66.7%)	0.696
Brain stem	0 (0.0%)	4 (100%)	0.230
Intraventricular	0 (0.0%)	1 (100%)	0.542
Operation			
Primary surgery	80 (25.0%)	240 (75.0%)	0.391
Reoperation	62 (28.3%)	157 (71.7%)	
Previous radiotherapy			
Yes	44 (31.4%)	96 (68.6%)	0.116
No	98 (24.6%)	300 (75.4%)	
Previous chemotherapy			
Yes	32 (32.0%)	68 (68.0%)	0.349
No	110 (25.1%)	329 (74.9%)	
Diabetes type 1 or 2			0.700
Yes	7 (23.3%)	23 (76.7%)	
No	135 (26.5%)	374 (73.5%)	
Cerebrovascular disease			0.024
Yes	10 (47.6%)	11 (52.4%)	
No	132 (25.5%)	386 (74.5%)	
ASA grade			
1–2	101 (25.2%)	300 (74.8%)	0.298
3–4	41 (29.7%)	97 (70.3%)	
Preoperative KPS			
≥ 70	18 (23.7%)	58 (76.3%)	0.570
< 70	124 (26.8%)	339 (73.2%)	
Surgeon			
Resident	20 (23.0%)	67 (77.0%)	0.438
Consultant	122 (27.0%)	330 (73.0%)	

## Discussion

In this population-based study, we found that peritumoral infarctions as diagnosed by early postoperative DWI are seen following nearly half of the glioma operations. However, most infarctions are small, and median infarction volume in patients with infarctions was only 1.7 ml. Sector-shaped infarctions, or a combination of rim- and sector-shaped infarctions, were larger in volume than rim-shaped infarctions and were seen in approximately one in four patients. Postoperative sector-shaped or combined rim- and sector-shaped infarctions were more common in patients operated for tumors in the temporal lobe and in patients with known cerebrovascular disease. Age and perioperative bleeding were positively associated with larger infarction volumes, while rim-shaped infarctions were more common in younger patients.

Peritumoral infarctions range from very small, rim-like infarctions around the operation cavity to large infarctions that cover a major vascular territory. There is no agreement on the definition of peritumoral infarctions, and the definition used will affect the incidence of such ischemic lesions. Very small DWI abnormalities due to the use of hemostatic agents, small blood clots, and physiological postoperative signal changes are frequently encountered [15, 22]. In the present study, increased DWI signals that measured less than 3 mm in diameter were labeled “not significant”. Although cutoffs may be debated, we earlier found good inter-rater agreement for detecting radiological significant DWI abnormalities when using a pragmatic radiological classification [9].

Still, other studies have classified such minimal rim DWI abnormalities along the surgical cavity as significant, increasing the incidence of infarctions to almost 90% [2]. However, we would argue that such wide definitions are less informative for assessing risk of clinically significant infarctions. Regardless, diffusion restriction should be routinely assessed at the initial postoperative MRI, as the infarctions over time exhibit contrast enhancement, posing a potential challenge in distinguishing infarctions and progressive tumor growth/malignant transformation in subsequent follow-up MRI scans [15].

Previous studies have reached contradictory conclusions as to whether recurrent surgeries are associated with an excess risk of peritumoral infarctions [8, 5]. In a study of 109 operations, peritumoral ischemic lesions were reported in up to 80% of patients after recurrent glioma surgery [8]. In our study, which is the largest to date, we did not find any excess risk of ischemic lesions following reoperations. Thus, fear of peritumoral infarctions should not be a major factor to consider when weighting potential risks of surgery for recurrent glioma.

We found that glioma resections in the temporal lobes were associated with higher risk of ischemic lesions, in line with previous reports [5]. We defined insula as a part of the temporal lobe, and the many perpendicular M3-M4 vessels crossing the insula are a known surgical challenge, which might contribute to the higher risk of ischemic complications and sector-shaped DWI changes in this region. However, another study found no association between the incidence of new ischemic lesions and tumor location [8]. Interestingly, the map-based visualization of infarctions indicates that there are more infarctions around the horns of the lateral ventricles, corresponding to known watershed areas in the brain. The subgroup analysis also revealed a higher rate of sector-shaped infarctions in patients with known cerebrovascular disease, indicating that patient-related vulnerability matters.

While high-grade and low-grade gliomas are very different with respect to (neo)vascularization, it is interesting that the risk of peritumoral infarctions was not associated with histopathology. Thus, the abundance of pathological vessels or edema does not seem to be associated with increased risk of infarctions in normal tissue. Still, increased perioperative bleeding may make it more difficult for the surgeon to separate normal and pathological arteries and ultimately increase the risk of infarctions.

A recent study found an association between persisting neurological deficits and clinically significant DWI changes after resection of diffuse low-grade gliomas [24]. These findings indicated that peritumoral infarctions may be a more common cause of deficits following low-grade glioma surgery than surgical resection of functional brain tissue. Thus, the presence of peritumoral infarctions could have potential as a radiological quality measure in addition to extent of resection following glioma surgery. Studying the clinical impact of peritumoral infarctions is challenging and beyond the scope of this study. Neurological deficits are location-dependent, and infarctions may often occur in areas where loss of function can be difficult to measure. No measured deficits may not be the same as no deficits, as patients may still suffer from less visible or assessable deficits like fatigue, reduced cognitive functions, psychological distress, altered personality, or impaired executive functions. Thus, studying a potential detrimental effect on peritumoral infarctions should perhaps involve neuropsychological testing or measures of health-related quality of life, in addition to conventional neurological deficits and survival [14]. Further research on the long-term effects of peritumoral infarctions on neurological deficits is warranted.

It is still not clear what measures can be taken to minimize the risk of infarctions in relation to tumor surgery. In a study exploring the relationship between perioperative hemodynamics, postoperative infarctions, and overall survival, diastolic blood pressure, a positive liquid balance, and duration of surgery were associated with postoperative infarction volumes [3]. Our data suggest a correlation between perioperative bleeding and infarction volume. However, duration of surgery was not associated with larger infarction volumes or excess risk. This could suggest that both surgical technique and close perioperative anesthesiologic monitoring could be a key to minimize the risks of peritumoral infarctions. The increased risk with age might reflect age-dependent vascular vulnerability due to general vascular disease. The higher rate of sector-shaped infarctions in patients with known cerebrovascular disease and the heatmap showing more infarctions in the watershed areas support that vulnerability of the patients varies.

There are several factors beyond excessive resection of functional brain tissue surrounding the tumor that may cause deficits following surgery, including surgical hematomas, brain infarctions, contusions from spatulas, and postoperative infections. However, the relative importance of these factors is unknown. Several studies emphasize the importance of infarction volume [2–4], but even small ischemic lesions in eloquent locations may result in severe neurological sequelae [18]. Further, little is known about the risk of ischemic lesions in relation to surgical technique, e.g., use of suction vs. ultrasonic aspirator, subpial dissection vs. transsulcal approaches, outside-in vs. in-side-out resections, and more. Moreover, it is unknown if the risk of ischemia is surgeon-dependent, or if there is a learning curve. We did not find a significant difference in the incidence of postoperative infarctions between residents and consultant neurosurgeons in the present study. However, in a post-hoc analysis, we found different rates of infarctions and difference in infarction volumes when comparing individual surgeons, indicating that surgical skill or technique may be of importance. However, different surgeons with different degrees of experience and skill operate different tumors, and such comparisons are not necessarily fair. Future studies on the variations of surgical techniques in relation to infarction rates are of interest, but a radiologically and clinical focus on peritumoral infarctions may be of benefit on its own. Experienced glioma surgeons may perhaps remember how implementation of routine, early postoperative MRIs facilitated learning and calibrated their own techniques to obtain better resection grades. Reviewing the DWI sequence carefully after surgery may also be of value to learn and reduce risks in future patients.

The main strengths of the current study are the large sample size, population-based case selection, prospective collection of many data variables, and quantitative analyses based on volumetric segmentation. To the best of our knowledge, this is the largest study reporting the incidence and volumetric segmentations of peritumoral infarctions after glioma surgery. However, false-positive findings are possible in this explorative setting, as we assessed various risk factors for infarctions, without adjusting for multiple testing. Our single-center design could limit the external validity, as surgical treatment for gliomas varies from department to department, both in terms of indications, use of tools, and surgical techniques. Furthermore, infarctions with hemorrhagic transformation may have been falsely scored as no infarctions, as DWI abnormalities which also had hyperintense areas on T1-weighted series were not interpreted as infarctions.

## Conclusions

In this population-based cohort study, we found that peritumoral infarctions occurred in 44% after diffuse glioma operations. Infarctions were not more common following recurrent surgeries as compared to primary operations, but infarctions were more common in patients operated for tumors in the temporal lobe. The map-based visualization of infarctions indicates that there are more infarctions around the horns of the lateral ventricles, corresponding to known watershed areas in the brain. Increasing age, larger tumors, and more intraoperative bleeding were factors associated with larger infarction volumes. Infarction rates and infarction volumes differ across individual surgeons.
